# Depression and hypertension awareness, treatment, and control in a multiethnic population in the Netherlands: HELIUS study

**DOI:** 10.1007/s11739-021-02717-9

**Published:** 2021-04-03

**Authors:** Florence Fernald, Marieke Snijder, Bert-Jan van den Born, Anja Lok, Ron Peters, Charles Agyemang

**Affiliations:** 1 Department of Public Health, Amsterdam Public Health Research Institute, Amsterdam University Medical Centers, Meibergdreef 9, 1105 AZ Amsterdam, The Netherlands; 2 Department of Clinical Epidemiology, Biostatistics and Bioinformatics, Amsterdam Public Health Research Institute, Amsterdam University Medical Centers, Meibergdreef 9, 1105 AZ Amsterdam, The Netherlands; 3 Department of Internal and Vascular Medicine, Amsterdam University Medical Centers, Meibergdreef 9, 1105 AZ Amsterdam, The Netherlands; 4 Department of Psychiatry, Amsterdam University Medical Centers, Meibergdreef 9, 1105 AZ Amsterdam, The Netherlands; 5 Department of Cardiology, Amsterdam University Medical Centers, Meibergdreef 9, 1105 AZ Amsterdam, The Netherlands

**Keywords:** Significant depressed mood, Depression, Hypertension, Ethnic groups, The Netherlands, HELIUS study

## Abstract

Individuals belonging to ethnic minority groups are more susceptible to depression and comorbid hypertension than European host populations. Yet, data on how depression is related to hypertension in ethnic groups in Europe are lacking. Therefore, we studied the association between significant depressed mood (SDM) and hypertension prevalence, awareness, treatment, and control among ethnic groups. Data from the HELIUS study included 22,165 adults (aged 18–70) from six ethnic backgrounds in the Netherlands. Logistic regression analysis was used to explore the association between SDM and hypertension prevalence, awareness, treatment, and control with adjustment for age, sex, and for sensitivity analysis purposes also for anti-depressants. After adjustment for age and sex, Dutch with SDM had an increased odds of hypertension (OR 95% CI 1.67; 1.08–2.59). Among Turkish, SDM was associated with higher odds of hypertension awareness (2.09; 1.41–3.09), treatment (1.92; 1.27–2.90) and control (1.72; 1.04–2.83). Among Moroccans, SMD was associated with an increased odds of hypertension awareness (1.91; 1.14–3.21) but decreased odds of hypertension control (0.42; 0.20–0.89). Additional adjustment for anti-depressant medications did not change the results. There were no associations between SDM and hypertension, awareness, treatment and control in South-Asian Surinamese, African Surinamese and Ghanaian participants. The results underline significant differences in the association between SDM and hypertension awareness, treatment and control between ethnic groups. Our findings emphasize the necessity to further study ethnicity-related factors that may influence the association between SDM and hypertension to promote hypertension control especially, among Moroccans with SDM.

## Introduction

It is well described that depression is a common risk factor for hypertension [1, 2] and consequent risk of cardiovascular disease (CVD) [3]. Stress experienced by individuals with depression has the potential to cause dysregulation in overactivation of the nervous system and hyperactivity of hypothalamic–pituitary–adrenal axis, thereby increasing the risk of hypertension [4]. Additionally, depression and hypertension share common disease pathways, possibly negatively affecting one another [2]. Apart from the link between pathophysiological pathways, poor adherence to anti-hyppertensive drugs [1, 5] and inadequate detection of depression in primary care settings [1] also inhibit blood pressure (BP) control among depressed individuals. Additionally, unhealthy lifestyle behaviors (e.g., physical inactivity) are more common among depressed individuals [6] and are another reason for hypertension [7]. However, evidence also shows that use of anti-depressants like tricyclic anti-depressants (TCAs) and noradrenergic and serotonergic (NS) working anti-depressants increase the risk of hypertension [8].

Besides, the susceptibility to hypertension and depression among ethnic groups differs due to various ethnic-related factors. For example, genetic markers for hypertension [9], cultural beliefs towards hypertension [10] and depression [11] (e.g., perception of anti-hypertensive [12] and anti-depressant treatment [11, 13]) and nonadherence with medication regimes vary across ethnic groups [14]. These differences may also impact hypertension management in ethnic groups differently.

The prospect of a further rise of hypertension [15], increase in the burden of depression [16], and consequent CVD and their impact on the individual and social–economic level, makes understanding of multimorbidity including mental health conditions a necessity as it is associated with an increased risk of premature mortality [17]. However, data on how depression relates to hypertension awareness, treatment and control across various ethnic groups in Europe are lacking, despite the fact that individuals belonging to ethnic minority groups are found to be more susceptible to depression [11] and hypertension [12] than European host populations. Therefore, we studied ethnic differences in the association between significant depressed mood (SDM) and hypertension prevalence, awareness, treatment, and control. We hypothesized that hypertension awareness, treatment and control are less likely in people with SDM, particularly in the ethnic minority groups, and that the association of SDM with hypertension awareness, treatment, and control of hypertension varies across ethnic groups, possibly due to differences in, amongst others, cultural beliefs.

## Methods

### Study design

For the analyses, baseline data from the HELIUS (HEalthy LIfe in an Urban Setting) study were used. Conducted by the Amsterdam University Medical Centres (Amsterdam UMC) and the Public Health Service of Amsterdam, the HELIUS study focuses on health and health-care utilization among different ethnic groups living in Amsterdam, the Netherlands. The aims and design of the large-scale prospective cohort study have been described in detail elsewhere [18, 19]. In brief, the study concentrates on three major disease categories including cardiovascular disease, mental health and infectious diseases, and focusses on people aged 18–70 years from the six major ethnic groups in Amsterdam, i.e., those of African Surinamese, South-Asian Surinamese, Turkish, Moroccan, Ghanaian, and Dutch origin.

After approval of the study protocols by the Amsterdam UMC Ethical Review Board and written informed consent of the participants, a baseline of 22,165 participants were collected by questionnaire and a physical examination between January 2011 and November 2015 [18].

For the current analyses, participants with unknown/other ethnic origin (*N* = 48), Javanese Surinamese origin (*N* = 233) and unknown/other Surinamese origin (*N* = 267), were excluded from the analysis due to relatively small numbers. In addition, subjects with missing data on hypertension and/or SDM (*N* = 254) were excluded. This resulted in a dataset of 21,363 participants including 4550 Dutch, 4104 African Surinamese, 3017 South-Asian Surinamese, 3558 Turks, 3853 Moroccans, and 2281 Ghanaians.

### Definitions and measurements

The country of birth of the participant as well as that of his/her parents was used to define the participant’s ethnic origin [20]. More specifically, a person is defined as of non-Dutch ethnic origin (ethnic minority) if the participant fulfilled either of the following criteria: (1) s/he was born abroad and has at least one of her/his parents born abroad; or (2) s/he was born in the Netherlands but has both his/her parents born abroad. For the Dutch sample, people who were born in the Netherlands and whose parents were born in the Netherlands were invited. Given that the Surinamese population is made up of several ethnic subgroups, self-identification (by questionnaire) was used to differentiate Surinamese origin subgroups into ‘African’, ‘South-Asian’, ‘Javanese’ or ‘other/unknown’ Surinamese origin. Information on the migration history of participants with a Surinamese, Ghanaian, Turkish, Moroccan background has been described in more detail elsewhere [18].

Educational level was based on the highest qualification obtained either in the Netherlands or in the country of origin and was categorized into four groups; those who have never been to school or had elementary schooling only, those with lower vocational schooling or lower secondary schooling, those with intermediate vocational schooling or intermediate/higher secondary education schooling, and those with higher vocational schooling or university.

Weight was measured in light clothing with SECA 877 scales to the nearest 0.1 kg. Height was measured without shoes with a portable stadiometer (SECA 217) to the nearest 0.1 cm. Body mass index (BMI) was calculated as weight (kg) divided by height squared (m^2^). Blood pressure (BP) was measured using an automated digital BP device (Microlife WatchBP Home, Microlife AG, Widnau, Switzerland) on the left arm in a seated position after the subject had been seated for at least 5 min. Anthropometric and BP measurements were performed in duplicate; the mean of the two measurements was used in the analyses.

Hypertension was defined as systolic BP ≥ 140 mmHg, or diastolic BP ≥ 90 mmHg, or using BP lowering medication [21]. Awareness of hypertension was defined as the proportion of hypertensive individuals who self-reported any prior diagnosis of hypertension by a health-care professional. Treatment of hypertension was defined as the proportion of hypertensive individuals who were receiving prescribed anti-hypertensive medication for high BP management. BP control was defined as the proportion of hypertensive individuals on anti-hypertensive medication with systolic BP < 140 mmHg and diastolic BP < 90 mmHg.

Depressive symptoms were measured by the 9-item Patient Health Questionnaire (PHQ-9) [22]. Its cross-cultural validity has been demonstrated across the ethnic groups included in the HELIUS study [23]. The PHQ-9 consists of nine items, with a response scales 0 ‘not at all’, 1 ‘on several days’, 2 ‘on more than half of the days’ and 3 ‘nearly every day’. If one of the 9 items was missing, the mean score of the other eight items was used to replace the missing item. If more than one item was missing, the variable was considered missing. A participant was considered to have a significant depressed mood (SDM) when one or both of items 1 (little interest or pleasure in doing things) and 2 (feeling down, depressed, or hopeless) were answered with at least ‘on more than half of the days’, and at least 5 of the 9 items were answered with at least ‘on more than half of the days’. The final item (suicidal ideation) already counted if answered with ‘on several days’ [24].

All participants were asked to bring their prescribed medications to the research location and anti-depressant agents were identified and categorized using Anatomical Therapeutic Chemical (ATC) classification system: selective serotonin reuptake inhibitors (SSRIs) and serotonin–norepinephrine reuptake inhibitors (SNRIs) (ATC codes N06AB and N06AX), mood stabilizers (ATC codes N03AF, N03AG, N05AN, N03AX) and tricyclic anti-depressants (TCAs) (ATC code N06AA). Blood pressure lowering drugs included: centrally acting anti-hypertensives (ATC code C02), diuretics (ATC code C03), beta blockers (ATC code C07), calcium channel blockers (CCBs) (ATC code C08), and agents acting on renin–angiotensin system (RAS) (ATC code C09).

### Statistical analyses

The Statistical Package for the Social Sciences (SPSS) was used for data analysis. Percentages, means and their corresponding 95% confidence intervals (CIs) were used to explore the characteristics of the study population. Odds ratios and their corresponding 95% (CIs) were computed using logistic regression analyses with adjustment for age, sex. Given that anti-depressant use can cause increased blood pressure levels [8], and influence hypertension treatment and subsequently blood pressure control, we additionally adjusted for anti-depressant use in sensitivity analyses. The interaction between ethnicity and SDM was also studied to test the hypothesis that the association between SDM and hypertension prevalence, awareness, treatment and control was different for ethnic groups*.*

## Results

### Study population characteristics

The study population stratified by the presence of SDM is shown in Table 1. In the total study population, 7.7% (*N* = 1640) of the participants reported to have SDM. Ethnic minority groups were more likely than Dutch to report SDM with the highest prevalence in the Turkish (29.4%) and Moroccan populations (26.4%). In general, women were more likely to have SDM than men, and alcohol use and the educational level were lower among individuals with SDM compared to those without SDM. The opposite was true for smoking and being overweight. But across ethnic groups there were differences regarding the relation between SDM, education, BMI, alcohol use and smoking. In each ethnic group (except for the Ghanaian population) individuals with SDM had significantly lower educational levels (*P* ≤ 0.01) than their non SDM peers. Only Dutch and South-Asian Surinamese participants with SDM consumed less alcohol, while Ghanaians with SDM consumed more alcohol than their non SDM counterparts. However, the difference in the Ghanaian population was not significant (*P* = 0.43). Smoking was more prevalent among those with SDM, except for South-Asian Surinamese and Ghanaian people. Turkish and South-Asian Surinamese participants with SDM were more likely to be overweight (≥ 25 kg/m^2^) compared to their peers without SDM. BP levels were higher among people without SDM than those with SDM. Use of BP lowering medication was slightly higher among those with SDM. No significant difference (*P* = 0.63) in hypertension prevalence among those with and without SDM was observed. Among people with SDM, 14.1% reported to use anti-depressants, as opposed to 2.9% of the people without SDM.

**Table 1:** Population characteristics by significant depressed mood (SDM) status

	SDM (*n* = 1640)	No SDM (*n* = 19,723)	*P* value
Age (years)	43.9 (43.3–44.5)	44.3 (44.1–44.5)	0.28
Men (%)	35.4	42.8	< 0.001
Ethnicity (%)			< 0.001
Dutch	7.9	22.4	
South-Asian Surinamese	17.7	13.8	
African Surinamese	12.5	19.8	
Ghanaian	6.2	11.1	
Turkish	29.4	15.6	
Moroccan	26.3	17.3	
Educational level, ≥ intermediate vocational or intermediate/higher secondary (%)	42.8	57.2	< 0.001
Dutch	70.3	82.8	< 0.001
South-Asian Surinamese	39.9	53.6	< 0.001
African Surinamese	49.0	59.1	< 0.001
Ghanaian	24.0	31.7	
Turkish	35.1	45.0	< 0.001
Moroccan	46.5	51.7	< 0.05
Current smoking, yes (%)	32.8	23.2	< 0.001
Dutch	39.4	24.4	< 0.001
South-Asian Surinamese	32.1	27.9	
African Surinamese	45.8	31.0	< 0.001
Ghanaian	6.9	4.3	
Turkish	43.8	33.1	< 0.001
Moroccan	19.5	12.8	< 0.001
Alcohol use, yes (%)	34.7	52.2	< 0.001
Dutch	80.6	91.5	< 0.001
South-Asian Surinamese	49.1	57.3	< 0.001
African Surinamese	67.8	68.8	
Ghanaian	51.5	47.5	
Turkish	21.3	23.0	
Moroccan	6.7	7.5	
BMI (kg/m^2^)	28.3 (28.0–28.6)	27.0 (26.9–27.1)	< 0.001
BMI, overweight (≥ 25 kg/m^2^) (%)	69.2	60.6	< 0.001
Dutch	43.4	39.8	
South-Asian Surinamese	62.2	55.9	< 0.05
African Surinamese	69.6	66.3	
Ghanaian	76.2	74.2	
Turkish	78.2	71.3	< 0.05
Moroccan	69.7	65.8	
SBP (mmHg)	125.2 (124.3–126.0)	127.0 (126.8–127.3)	< 0.001
DBP (mmHg)	77.9 (77.4–78.4)	79.0 (78.8–79.1)	< 0.001
Hypertension	32.0	32.6	
BP lowering drug use^a^	18.8	16.4	< 0.001
Anti-depressant drug use^b^	14.1	2.9	< 0.001

Values are given as mean and corresponding 95% confidence interval or percentage

^a^BP lowering drugs include diuretics, beta blockers, centrally acting anti-hypertensives, calcium channel blockers and agents acting on the renin–angiotensin system

^b^Anti-depressant drugs include selective serotonin reuptake inhibitors (SSRIs), serotonin–norepinephrine reuptake inhibitors (SNRIs) and mood stabilizers and tricyclic anti-depressants (TCAs)

### Hypertension prevalence, awareness, treatment and control

**Fig. 1:**
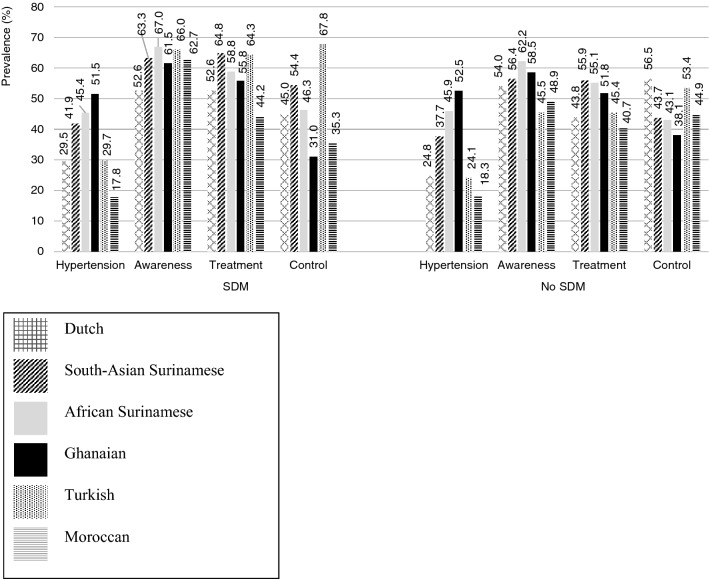
Proportional hypertension prevalence, hypertension awareness, treatment and control by significant depressed mood (SDM) status and ethnicity. Microsoft Excel was used to create Fig. . 1

Figure 1 shows the hypertension prevalence, awareness, treatment and control by SDM status and ethnicity. Regardless of SDM status, hypertension was most prevalent in the Ghanaian and African Surinamese populations. Among the Moroccan population, hypertension was the least prevalent (SDM 17.8%, SDM 18.3%). Irrespective of SDM status, African Surinamese were most aware of their hypertension status as opposed to individuals with other ethnic backgrounds. However, in contrast to other ethnic groups, hypertension treatment, was highest among South-Asian Surinamese people. Moroccan people were least treated for their hypertension (SDM 44.2%, no SDM 40.7%). BP control was more prevalent among people of Turkish origin as opposed to those with other ethnic backgrounds.

As shown in Fig. 1, among individuals with SDM, BP control was least prevalent in the Moroccan population (35.3%), while in the no SDM group Ghanaians had the lowest prevalence of BP control (38.1%).

Only Dutch individuals with SDM had a higher age–sex-adjusted odds of hypertension than their peers without SDM (OR 95% CI 1.67; 1.08–2.59, *P* = 0.02) (Table 2), although the association between SDM and hypertension lost its significance (*P* = 0.06) when anti-depressant drug use was further adjusted for (Table 3).

**Table 2:** The association (odds ratios with 95% confidence intervals) between SDM and hypertension, hypertension awareness, treatment and control by ethnicity

	Hypertension prevalence	Awareness	Treatment	Control
	OR (95% CI)	OR (95% CI)	OR (95% CI)	OR (95% CI)
Dutch	1.66 (1.08–2.56)*	1.02 (0.53–1.99)	1.71 (0.87–3.38)	0.57 (0.23–1.42)
South-Asian Surinamese	1.14 (0.86–1.52)	1.31 (0.87–1.98)	1.45 (0.96–2.21)	1.50 (0.91–2.35)
African Surinamese	0.99 (0.73–1.36)	1.13 (0.71–1.80)	1.02 (0.65–1.60)	1.03 (0.59–1.80)
Ghanaian	1.01 (0.64–1.59)	1.15 (0.64–2.08)	1.23 (0.69–2.18)	0.82 (0.36–1.84)
Turkish	1.17 (0.92–1.48)	2.09 (1.41–3.09)^*^	1.92 (1.27–2.90)^*^	1.72 (1.04–2.83)^*^
Moroccan	0,92 (0.70–1.43)	1.91 (1.14–3.21)^*^	1.26 (0.75–2.10)	0.42 (0.20–0.89)^*^

Adjusted for age and sex

^*^Significant association (*P* < 0.05)

After adjustment for age and sex, a positive association between SDM and hypertension awareness was significant for Turkish (2.09; 1.41–3.09, *P* < 0.001) and Moroccan (1.91; 1.14–3.21, *P* = 0.01) people only (Table 2). Additional adjustment for anti-depressants did not change the significance of the associations (*P* = 0.001 and *P* = 0.01, respectively) (Table 3). SDM was significantly associated with hypertension treatment only in Turkish people (1.92; 1.27–2.90, *P* = 0.002). Adjustment for anti-depressants attenuated the difference slightly (1.89; 1.24–2.87, *P* = 0.003). SDM was positively associated with hypertension control among Turkish people (1.68; 1.02–2.79, *P* = 0.04) only. The opposite result was found in the Moroccan population in which SDM was associated with decreased odds of hypertension control (0.39: 0.18–0.84, *P* = 0.02). Ethnicity was a significant effect modifier in the association between SDM and hypertension control (the association significantly differed between Turkish and Dutch people (*P* interaction = 0.04).

## Discussion

### Key findings

We studied associations between SDM and prevalence, awareness, treatment, and control of hypertension across ethnic groups. Our study shows that Turkish people with SDM were more likely to be aware of, treated and controlled for their hypertension compared with their Turkish counterparts without SDM. Moroccan people with SDM were also more likely to be aware of their hypertension status but were less likely to achieve adequate BP levels compared with their counterparts without SDM. There was no association between SDM and hypertension prevalence, awareness, treatment, and control among the other ethnic groups.

### Discussion of the key findings

The presence of an association (*P* = 0.02) between SDM and hypertension in the Dutch population corresponds with the results of the studies that showed an association between depression and hypertension [25, 26], while other studies reported opposite results [27, 28].

Findings on the association between SDM and hypertension remain inconclusive; no [27, 28], positive [1, 29], and inverse [9, 30, 31] associations were found earlier. This partly may be related to differences in the analyzed depression severity. While the association between depression severity and hypertension has been less studied, longitudinal data show that a lower depression prevalence at baseline increases the risk of hypertension incidence as opposed to a higher depression prevalence at baseline [1, 31]. A possible explanation is that a lower depression prevalence may denote a more severe depression [31].

Contrary to our hypothesis, we found that SDM was associated with more hypertension awareness, treatment and control only in Turkish and Moroccan populations compared with their counterparts without SDM. While there is little evidence that explains the findings, several factors might have contributed to the results.

First, primary health-care use has been shown to be higher among Turkish and Moroccan people (aged ≥ 55 years) compared to Dutch and Surinamese people [32]. Possibly, more visits to the general practitioner increased the frequency of BP measurements (as part of clinical decision making), subsequent hypertension awareness and control in Turkish, and hypertension awareness in Moroccan populations. However, sensitivity analysis showed that only in Turkish people, the likelihood of receiving hypertension treatment was significantly (*P* = 0.003) higher if SDM was present. A reason underpinning this finding may be the health professionals’ awareness about the higher prevalence of depressive disorders and depressive symptoms among Turks compared to other ethnic groups [23, 33, 34]. The additional understanding that depression and uncontrolled hypertension are related [2] could have led to a higher odds of hypertension treatment in the Turkish population. Similarly, familiarity with depression and issues associated with its management in the Turkish community could have had a positive effect on hypertension control in Turks with SDM. But this theory has yet to be proven.

The positive association between SDM and hypertension control in the Turkish population (*P* = 0.04) is in line with evidence that indicated that hypertension control was more likely in people with depression than in people without depression [35]. However, literature on the association between SDM and hypertension control is inclusive [2]. For example, depression is a known risk factor for poor adherence to anti-hypertensive medication [5], which lowers the chance of hypertension control.

We found a lower odds of hypertension control in Moroccan people with SDM (*P* = 0.02). Explanatory reasons for opposite associations between SDM and hypertension control in the Turkish and Moroccan population are unclear but may be related to the low prevalence of hypertension among Moroccans compared to other ethnic minority groups in the Netherlands [36]. Because hypertension is less common in the Moroccan community, it is possible that they receive less attention on preventive measures, e.g., adherence to treatment, which may contribute to poor hypertension control compared with other ethnic groups.

Additionally, an increased risk of becoming non-adherent to anti-hypertensive medication due to depression [5] could be mediated by low self-efficacy expectations [37] and a decreased desire and ability to follow treatment recommendations [38]. As a result, these factors might also have negatively affected hypertension control among individuals with SDM.

### Strengths and limitations

Two major strengths of our study were the large sample size and the inclusion of participants with different ethnic backgrounds, which allowed ethnicity-stratified analyses. Previous non-response analyses have shown that socioeconomic differences between participants and non-participants in HELIUS were very small [18]. It is expected that the European population, including the Netherlands [39, 40], will continue to become more ethnically diverse. For this reason, studies that reflect a diverse society are essential to provide inclusive health care. Moreover, regarding the association between SDM and hypertension management, our study is the first to examine association within different ethnic groups.

However, this study also has limitations. First, the cross-sectional design of this study challenged examination of cause–effect relationships between hypertension management and SDM. Second, self-report of SDM and hypertension awareness increased the risk of reporting bias. For instance, suggested ethnic differences in the association between stigma and SDM [15] could have led to under-report of SDM and consequent underestimation of the results.

In conclusion, this study shows ethnic variability in the association between SDM and hypertension treatment and control, i.e., only in Turkish and Moroccan groups associations were found for hypertension awareness, treatment and control. Other studies that explore these relationships among different ethnic groups are lacking. Therefore, research to better understand mediating factors to ethnic variations in the association of SDM with hypertension management outcomes is recommended to promote hypertension control and related sequelae.

## Data Availability

The statistical package for the social sciences (SPSS) was used for data analysis.

## Appendix

See Table 3.

**Table 3:** The association (odds ratios with 95% confidence intervals) between SDM and hypertension, hypertension awareness, treatment and control by ethnicity

	Hypertension prevalence	Awareness	Treatment	Control
	OR (95% CI)	OR (95% CI)	OR (95% CI)	OR (95% CI)
Dutch	1.53 (0.98–2.38)	1.09 (0.55–2.15)	1.51 (0.75–3.05)	0.43 (0.16–1.13)
South-Asian Surinamese	1.10 (0.83–1.47)	1.24 (0.82–1.88)	1.40 (0.92–2.14)	1.46 (0.90–2.36)
African Surinamese	0.97 (0.71–1.33)	1.13 (0.71–1.79)	0.99 (0.63–1.55)	1.00 (0.58–1.76)
Ghanaian	1.00 (0.63–1.59)	1.18 (0.65–2.13)	1.24 (0.69–2.22)	0.78 (0.34–1.78)
Turkish	1.17 (0.92–1.49)	2.02 (1.36–3.01)^*^	1.89 (1.24–2.87)^*^	1.68 (1.02–2.79)^*^
Moroccan	0.88 (0.66–1.17)	1.93 (1.15–3.25)^*^	1.21 (0.72–2.03)	0.39 (0.18–0.84)^*^

Adjusted for age, sex and anti-depressant drug use

^*^Significant association (*P* < 0.05)
